# Supplementary material for: MCL-1 gains occur with high frequency in lung adenocarcinoma and can be targeted therapeutically
Source: Nat Commun. 2020 Sep 10;11:4527. doi: 10.1038/s41467-020-18372-1 (PMC7484793; doi:10.1038/s41467-020-18372-1)
Supplement: Supplementary file 1 — Supplementary Information [file 41467_2020_18372_MOESM1_ESM.pdf]

***MCL-1* gains occur with high frequency in lung adenocarcinoma and can be targeted therapeutically**

Munkhbaatar *et al.*

Supplementary Information

Supplementary Figures

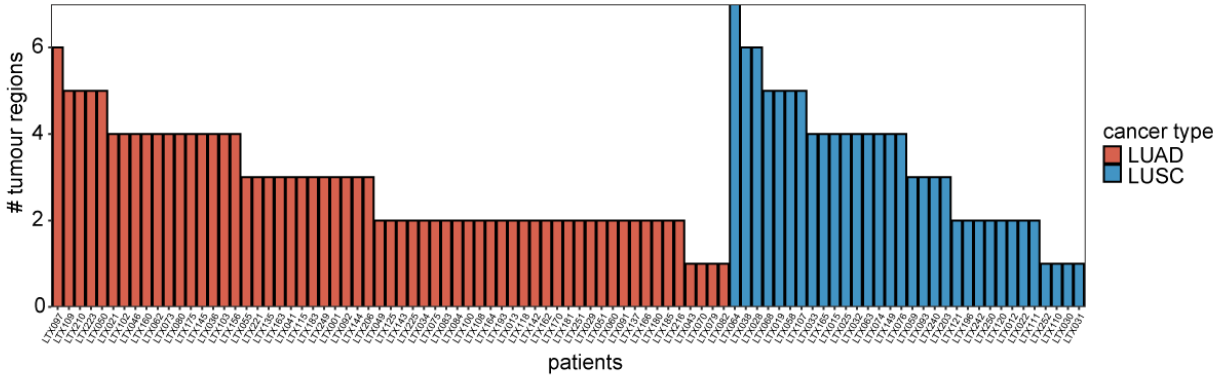

Supplementary Fig. 1 (related to main Fig. 1)
Number of tumour regions per patient of the multi-region whole exome sequencing study TRACERx. 61 patients (170 tumour regions) with lung adenocarcinoma (LUAD) and 32 patients (107 tumour regions) with lung squamous cell carcinoma (LUSC) were included in the analysis.

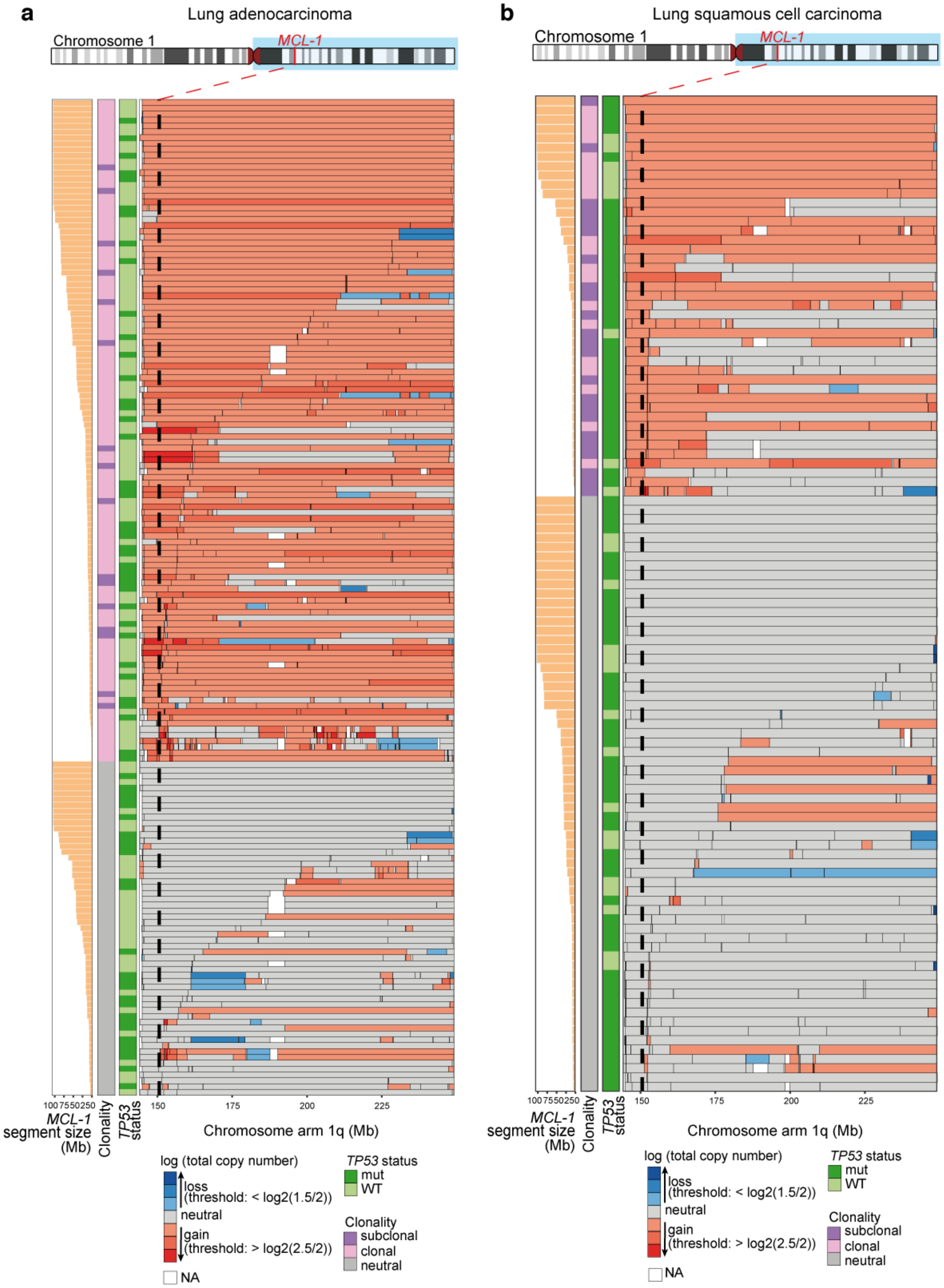

Supplementary Fig. 2 (related to main Fig. 1)
Overview of the segments located on chromosome arm 1q of TRACERx LUAD (a, n=170 tumour regions of 61 patients) and LUSC (b, n=107 tumour regions of 32 patients). Different shades of red indicate increasing copy number with a minimum of log2(2.5/2), which is the threshold for gains used in this analysis. Different shades of blue present decreasing copy number with a maximum of log2(1.5/2), the threshold for

losses. The dashed black line represents the position of *MCL-1*. The barplot on the left shows the size of the segment (in Mb) that *MCL-1* Is located on. In addition, in this figure we are reporting the *TP53* status (dark green, mutant; light green, wild type) and the *MCL-1* gain clonality (purple, subclonal; pink, clonal).

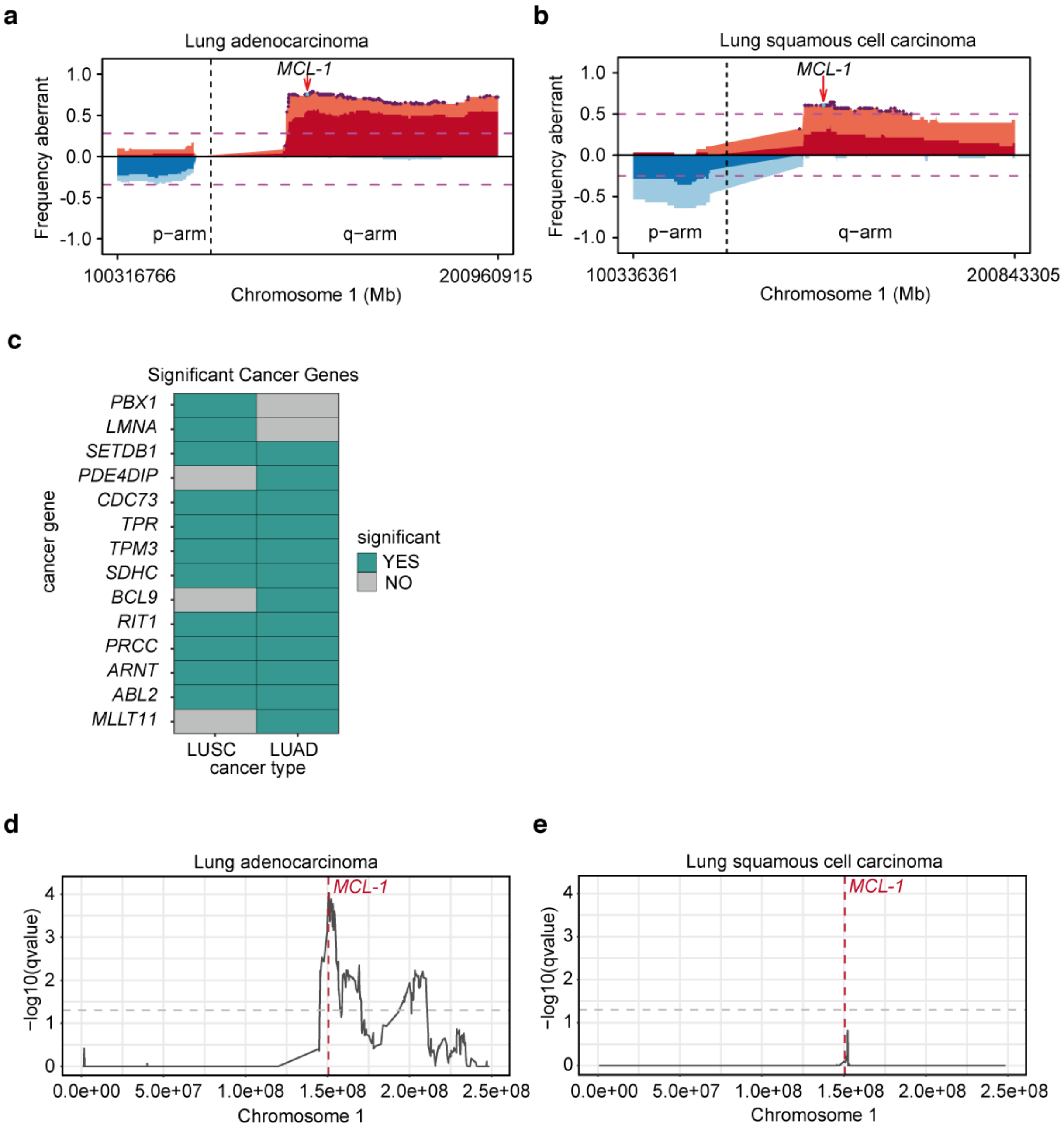

Supplementary Fig. 3 (related to main Fig. 1)
(a-b) Location and distribution of genes in the 1q21 region, whose mRNA levels are significantly upregulated ($p<0.05$, one-sided Wilcoxon-test) with a gain of the corresponding genes in TRACERx LUAD (a, n=61) and LUSC (b, n=32) samples. (c) Heatmap of significant putative cancer genes on the 1q21 amplicon for LUAD and LUSC, based on published cancer driver lists. (d-e) GISTIC2 results for amplifications on chromosome 1 of TRACERx LUAD and LUSC samples. The mean copy number across regions within a tumour were used for each minimum consistent region segment. The negative log10 transformed q-values are displayed for the different segments located on chromosome 1. The vertical dashed red line highlights the position of *MCL-1* and the horizontal grey line indicates the cut-off for significance (alpha = 0.05). A significant peak of copy number amplifications can be detected in LUAD patients but not in LUSC.

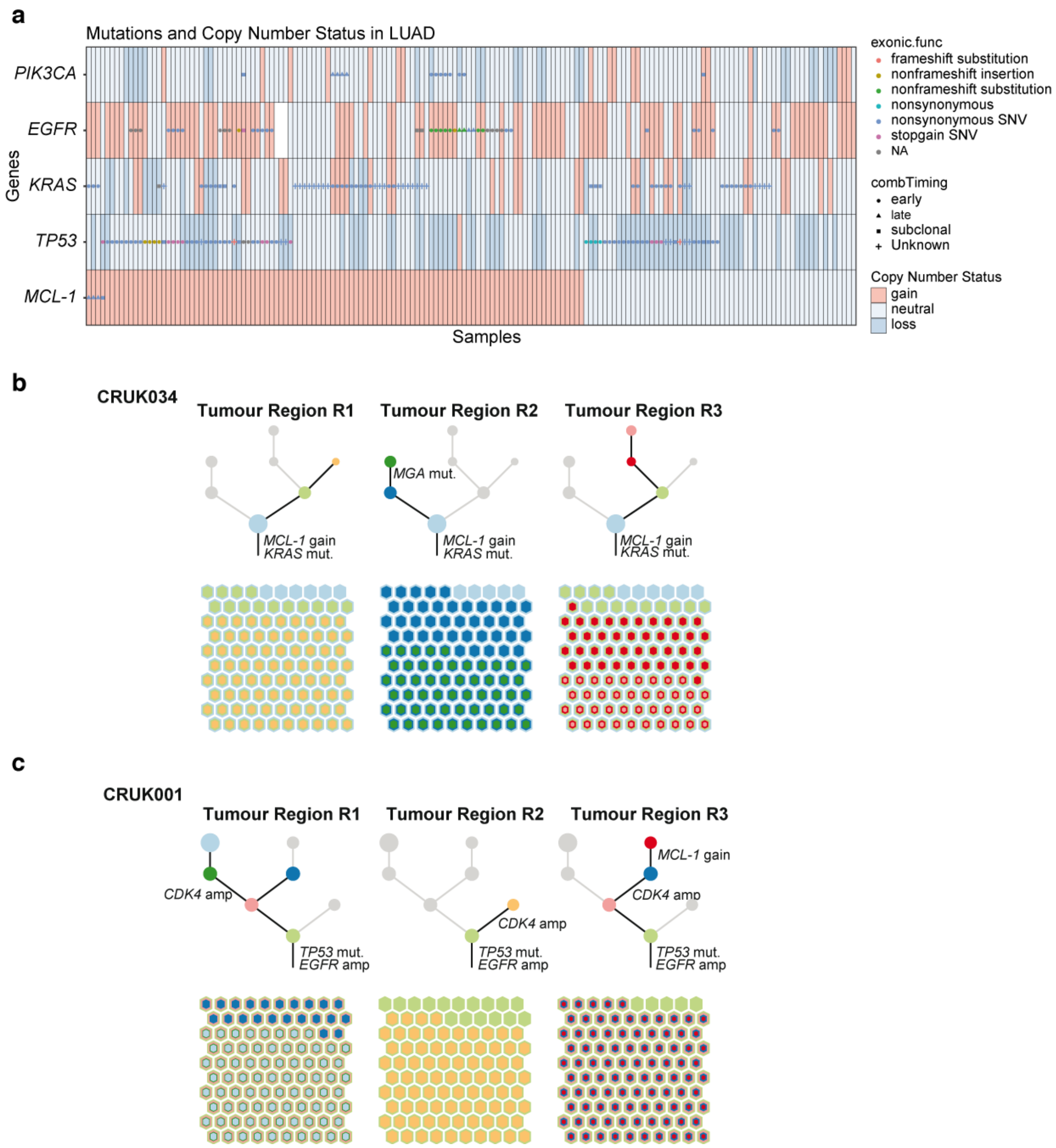

Supplementary Fig. 4 (related to main Fig. 2)

(a) Co-occurrence of *MCL-1* gain and mutations in lung cancer driver genes. The background colour of the different tiles indicates the copy number status of the different genes in the 170 LUAD tumour regions of the TRACERx study. Red presents copy number gain, blue copy number loss, and light blue no copy number change. Mutations are illustrated by points with different colour and shapes, where colour specifies different exonic functions and shape timing. (b) Phylogenetic trees depicting the clonal architecture of three individual tumour regions of patient CRUK001, with clusters not found in a given region shown in grey. Somatic driver events, like the clonal *TP53* mutation and *EGFR* amplification (light green) and the subclonal *MCL-1* gain (red) are annotated in the trees. The cellular prevalence of the subclonal clusters is shown as a grid of 100 representative cells beneath the trees in which nested colours within each

cell represent the mutational clusters present. (c) Phylogenetic trees depicting the clonal architecture of three individual tumour regions of patient CRUK034, with clusters not found in a given region shown in grey. *MCL-1* gain is identified as an event present in every cancer cell of the patient and therefore found in the clonal cluster (light blue). The cellular prevalence of the subclonal clusters is shown as a grid of 100 representative cells beneath the trees in which nested colours within each cell represent the mutational clusters present.

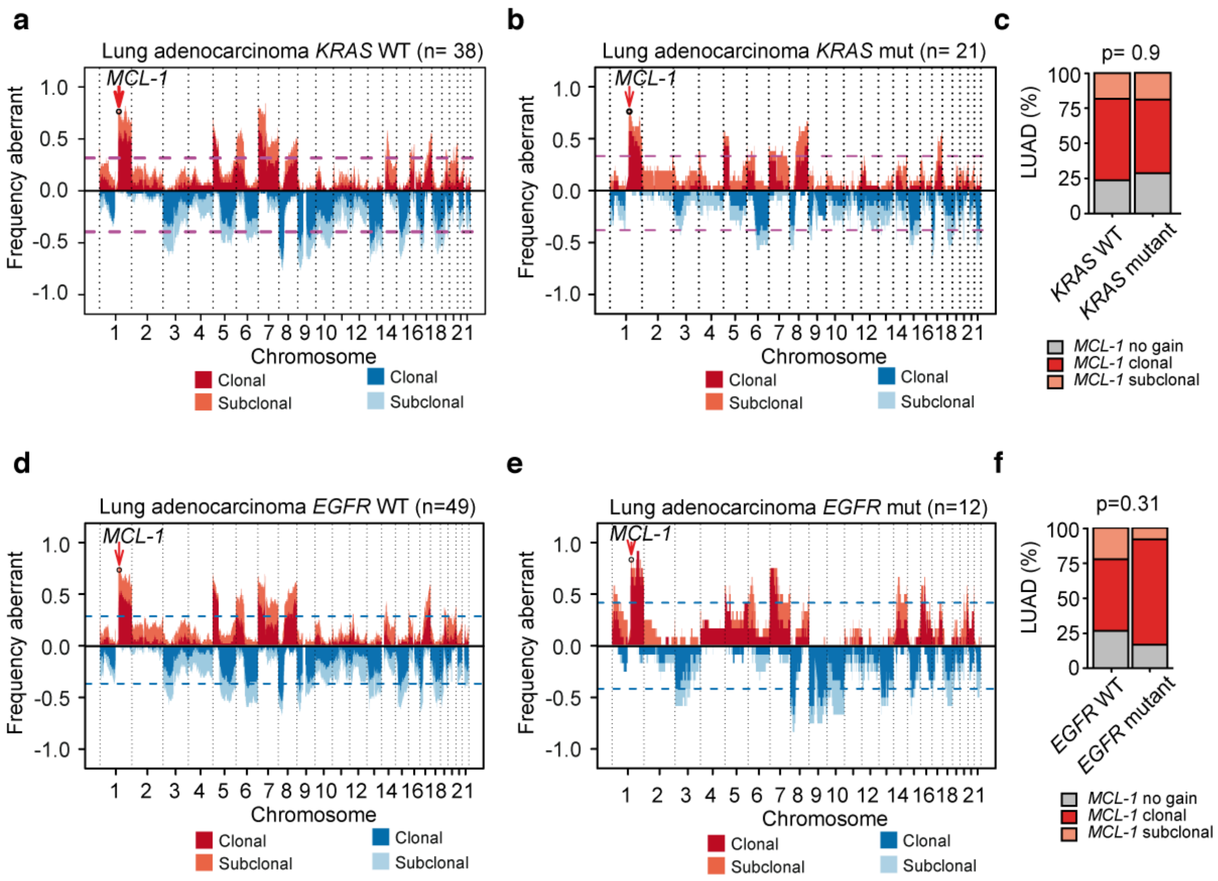

Supplementary Fig. 5 (related to main Fig. 2)

(a-b) Copy number aberrations expressed as frequency in TRACERx LUAD grouped for *KRAS* wild-type (WT) (a) and mutant (b). Shading indicates clonal status. n= number of patients. (c) Frequency distribution of *MCL-1* gain in *KRAS* WT or clonal mutant TRACERx LUAD samples. Data were analysed by Pearson´s chi-squared test with continuity correction (p=0.9, chi-square(2)=0.2). (d-e) Copy number aberrations expressed as frequency in TRACERx LUAD grouped for *EGFR* wildtype (WT) (d) and mutant (e). Shading indicates clonal status. n= number of patients. (f) Frequency distribution of *MCL-1* gain in *EGFR* WT or clonal mutant TRACERx LUAD samples. Data were analysed by Pearson´s chi-squared test with continuity correction (p=0.31, chi-square(2)=2.35).

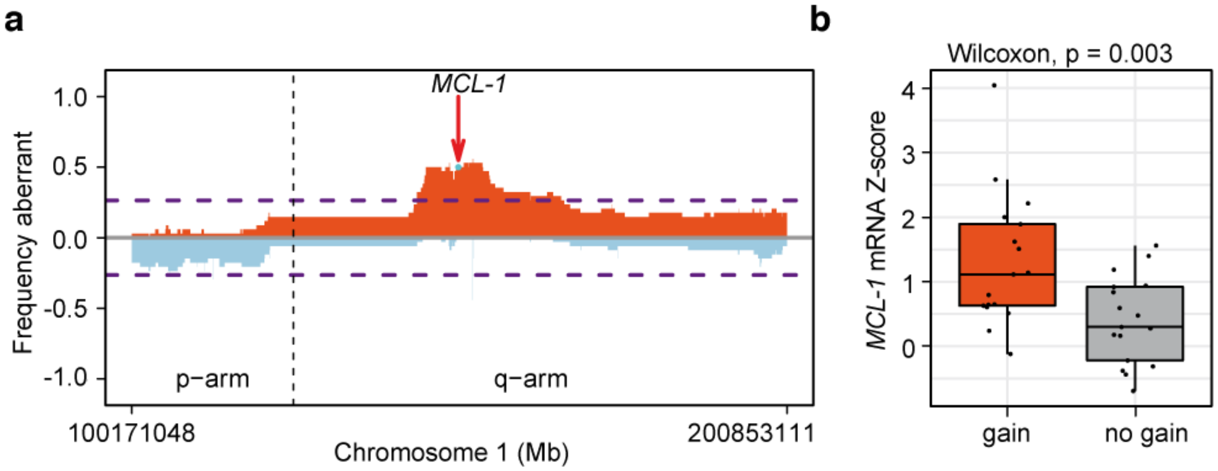

Supplementary Fig. 6 (related to main Fig. 4)

Copy number and expression data for LUAD cell-lines (n = 34) has been downloaded from the COSMIC Cell Lines Project. (a) The frequency of copy number gains (log2(2.5/2)) and losses (log2(1.5/2) has been calculated in the same way as for TRACERx and TCGA. The region around *MCL-1* shows a significant increase in copy number gains across the different cell lines. (b) A significant increase in *MCL-1* expression is present in cell lines with *MCL-1* gain in comparison to cell lines with no gain (as detected by one-sided Wilcoxon test, 17 gain and 17 no gain, p=0.003, W=223). Data are presented as box plot, where the centre line represents the median value, the limits represent the 25th and 75th percentile, the whiskers represent the minimum and maximum value of the distribution excluding the outliers defined using the inter-quartile range (IQR) rule. The points represent the individual experimental values.

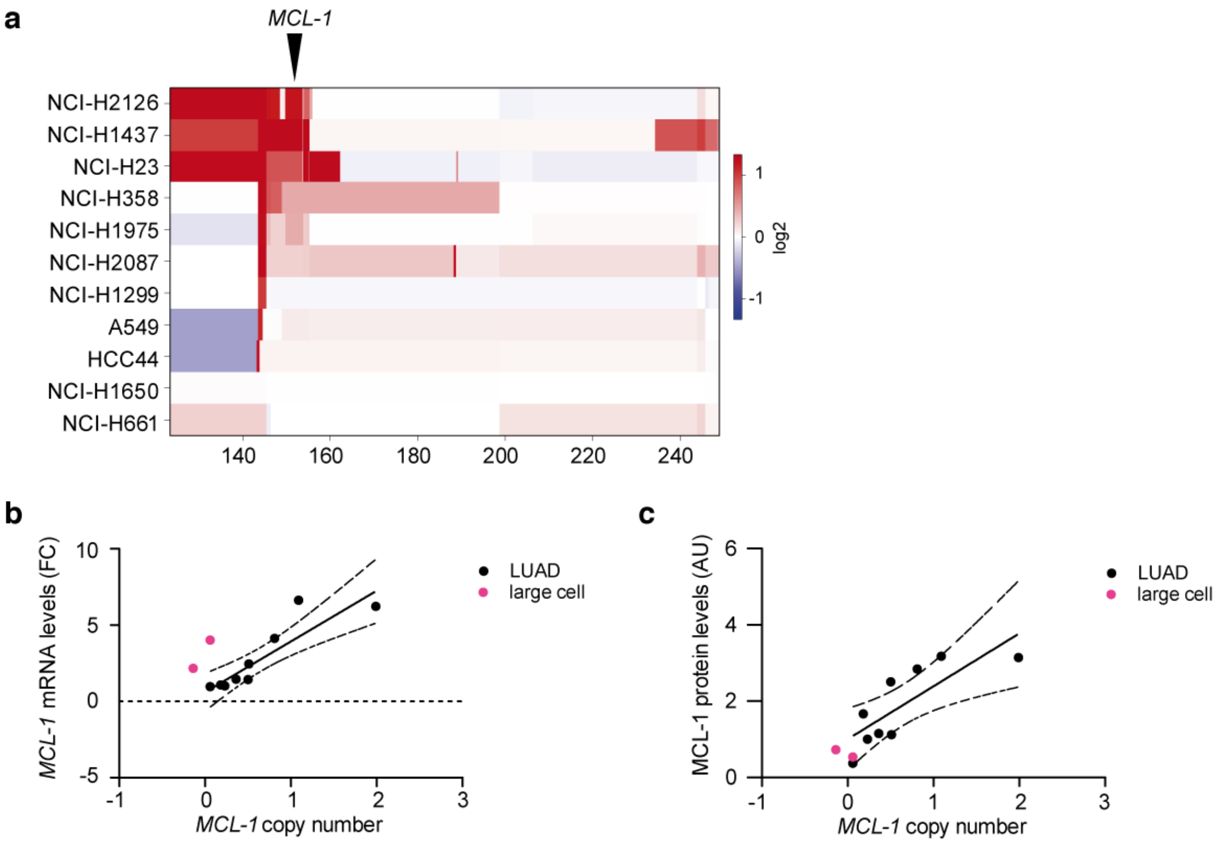

Supplementary Fig. 7 (related to main Fig. 4)

(a) Low coverage whole genome sequencing-based copy number profile of chromosome arm 1q for human lung cancer cell lines. (b-c) Linear regressions between *MCL-1* copy number and *MCL-1* mRNA levels (a, LUAD and large cell: R^2=0.7664 and p=0.0009; only LUAD: R^2=0.799 and p=0.0012) and protein levels (b, LUAD and large cell: R^2=0.6204 and p=0.0068; only LUAD: R^2=0.6304 and p=0.0010). In panel b-c the dashed lines represent the 95% confidence interval for the linear regression for only LUAD cell lines.

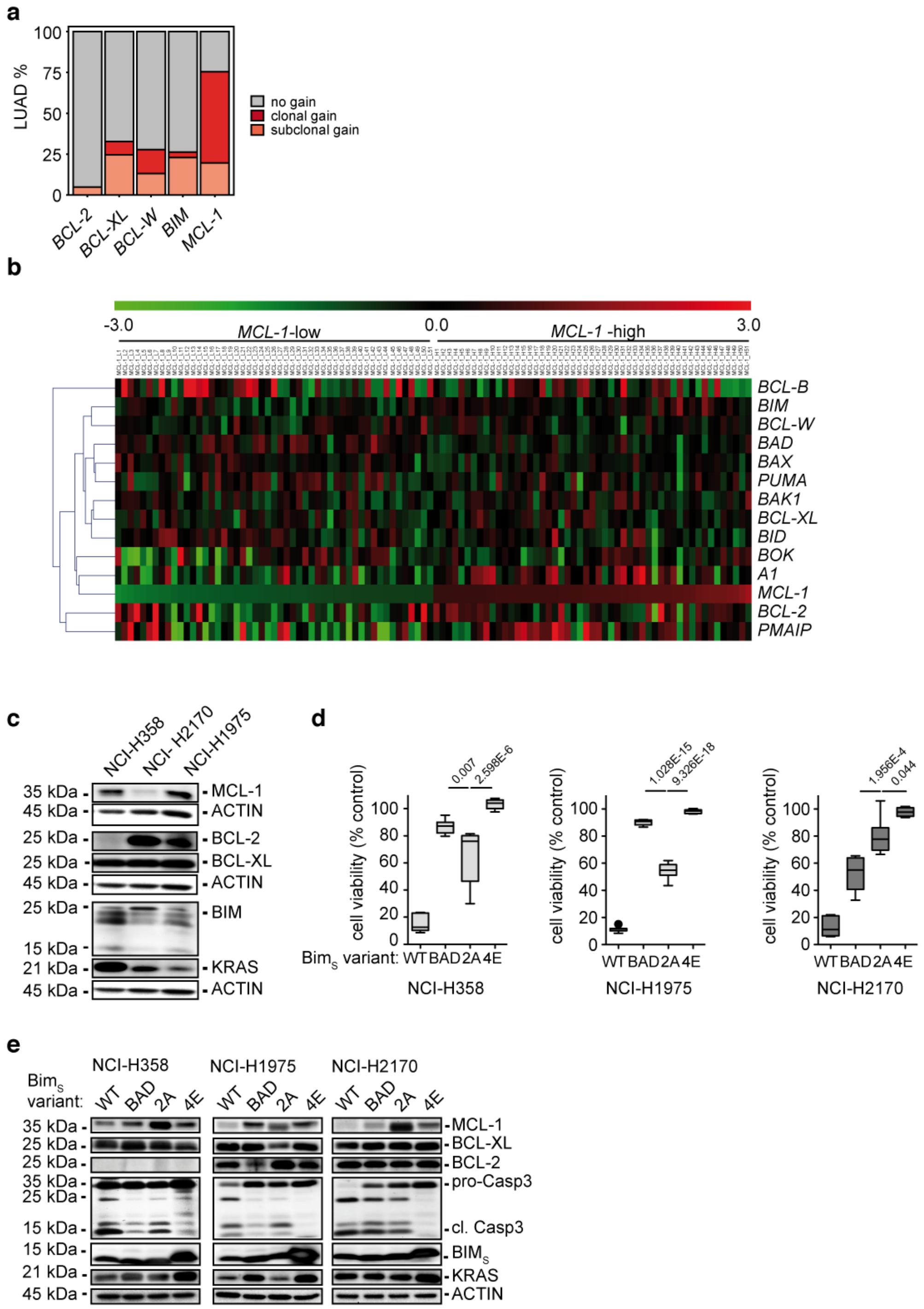

Supplementary Fig. 8 (related to main Fig. 4)

(a) Copy number gains expressed as frequency in TRACERx LUAD. In this figure we show analysis of the copy number gain of the BCL-2 family members: *BCL-2, BCL-XL, BCL-W, BIM,* and *MCL-1*. Shading indicates clonal status. n=61 number of patients. (b) Heatmap of *BCL-2* family gene expression in samples segregated into *MCL-1*-low *vs MCL-1*-high groups. The mRNA expression levels of *BCL-2* family members were clustered in both rows and columns before being plotted into a heatmap. The colour intensity indicates medium centralised expression level for each gene. (c) Immunoblotting to detect the indicated proteins in the NSCLC cell lines. Probing for β-actin served as a loading control. (d) Relative viability of the indicated cell lines 48 h after induction of the expression of the different BIM_S variants, as determined by FACS analysis. Percentage of viable untreated cells was assigned as 100% (one-way ANOVA: NCI-H358: $p=1.287E-14$, $F(3,28)=92.13$; NCI-H1975: $p=3.371E-25$,

F(3,24)=975.4; NCI-H2170: p=3.334E-12, F(3,22)=83.99). p values from post hoc analysis with Bonferroni correction are reported in the panel. Data are presented as box plot, where the centre line represents the median value, the limits represent the 25th and 75th percentile, and the whiskers represent the minimum and maximum value of the distribution. The points outside the whiskers were defined by the IQR rule. (e) Immunoblotting to detect the indicated proteins 24 h after the induction of the expression of the different BIM_S variants in the NSCLC cell lines. Probing for β-actin served as a loading control. Casp3: caspase-3. For data in panel c, d, and e, each cell line was assayed at least in 3 independent experiments.

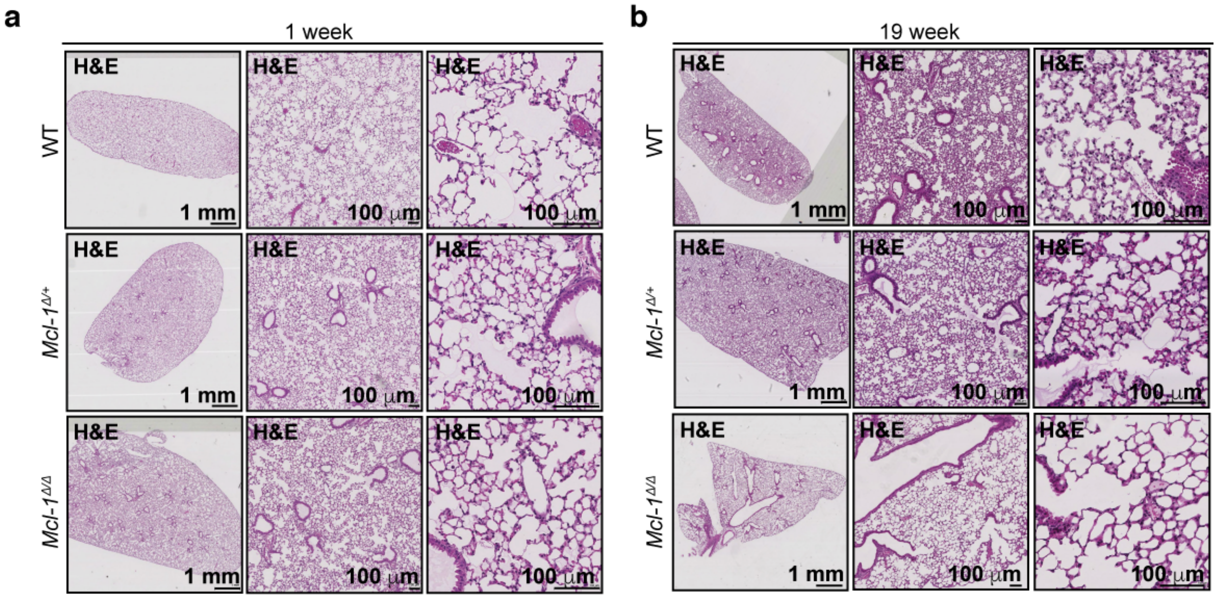

Supplementary Fig. 9 (related to main Fig. 5)
(a-b) Representative H&E staining of WT, *Mcl-1*$^{\Delta/+}$, and *Mcl-1*$^{\Delta/\Delta}$ lungs at one week (a) and 19 weeks (b) post-infection with AdCre. Different magnifications are shown (from right to left: 1x, 4x, and 20x). Scale bar is 1 mm or 100 μm. At least 3 mice per genotype were infected per time point in 6 independent experiments. All attempts at replication were successful.

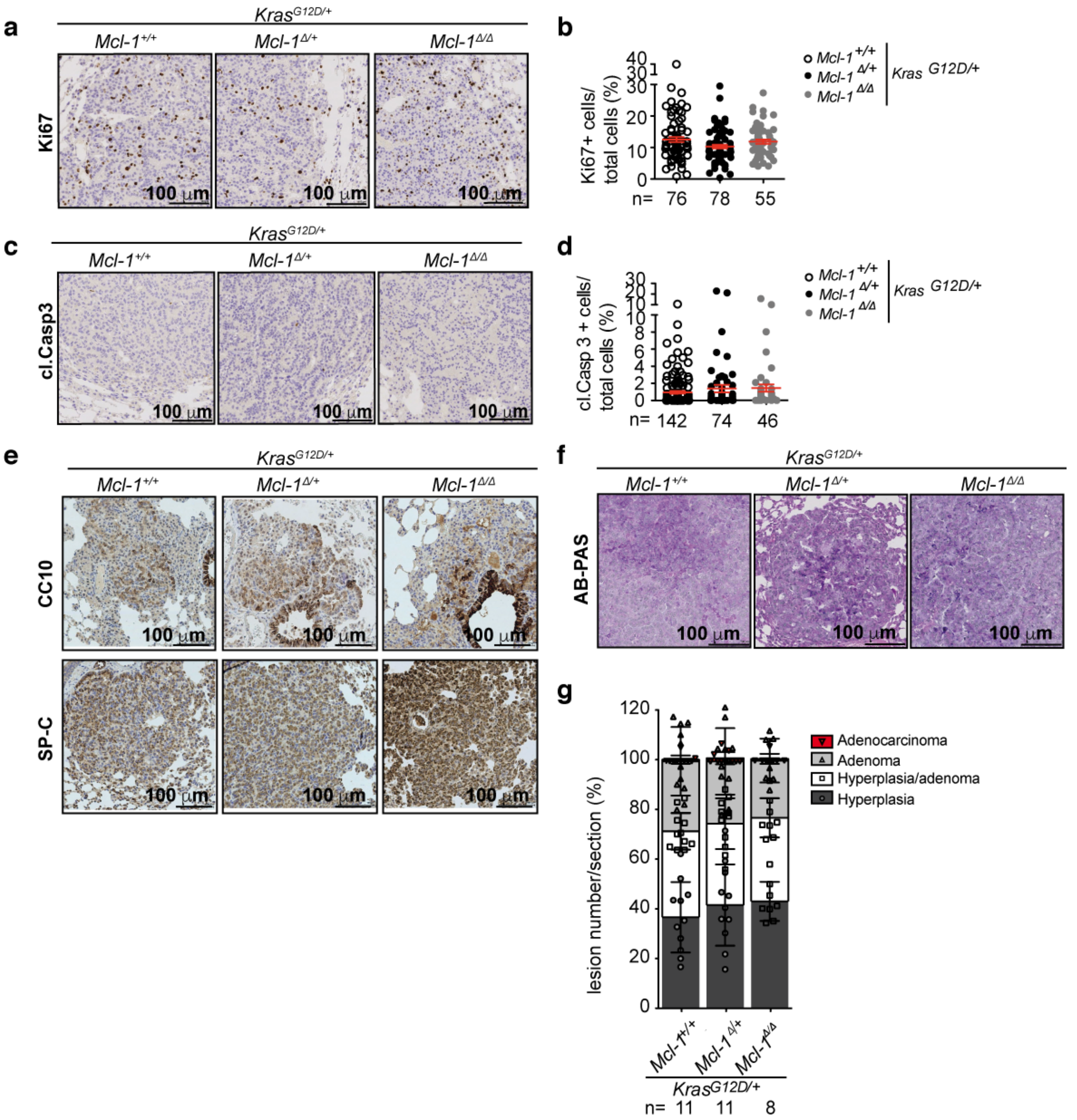

Supplementary Fig. 10 (related to main Fig. 5)

(a-b) Representative images (a) and quantification (b) of Ki67 positive cells (used as a marker of proliferation) in *Kras^G12D/+^ Mcl-1^+/+^*, *Kras^G12D/+^ Mcl-1^Δ/+^* and *Kras^G12D/+^ Mcl-1^Δ/Δ^* lesions. Six to eight lesions per section per mouse were analysed. Quantifications are represented as percentages of positive cells relative to all cells in the assessed lesions at 19 weeks post-infection with the AdCre virus. n=number of lesions. (c-d) Cleaved (i.e. activated) caspase-3 staining (marker of apoptosis) (c) and quantification (d). One section per mouse was analysed. All lesions within a section of a mouse were assessed. n=number of lesions. Data in panel b and d are presented as dot plots and report mean ± SEM. (e-f) CC10 and SP-C (e), and AB-PAS (f) staining of the *Kras^G12D/+^ Mcl-1^+/+^*, *Kras^G12D/+^ Mcl-1^Δ/+^* and *Kras^G12D/+^ Mcl-1^Δ/Δ^* lesions at 19 weeks post-infection with the AdCre virus. (g) Percentages of proliferative lesions of different classes from mice of the indicated genotypes at 19 weeks post-infection. One section per mouse was analysed. n=number of animals. The results shown are representative of 4 independent experiments. Data in panel g are presented as mean ± SD.

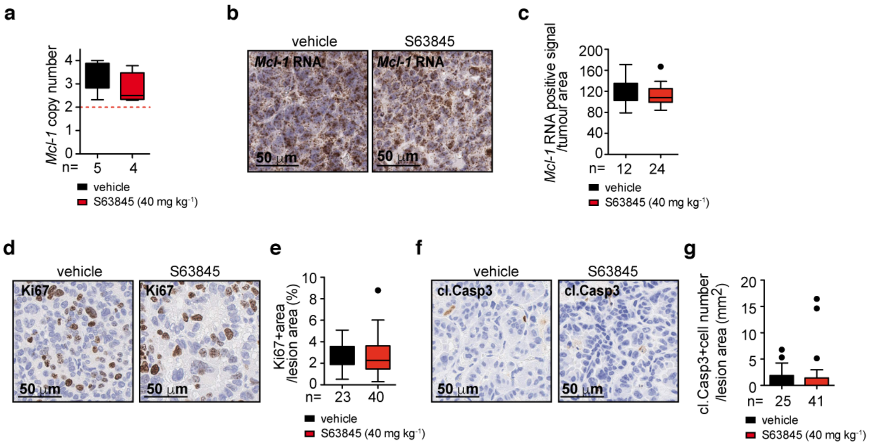

Supplementary Fig. 11 (related to main Fig. 6)

(a) *Mcl-1* copy number evaluated on gDNA extracted from FFPE lesions (2 lesions from at least 4 mice/group) by Taqman® assay using the CopyCaller® Software. n=number of mice. (b-c) Representative images (b) and quantification (c) of *Mcl-1* mRNA *in situ* hybridisation in lesions from vehicle- and S63845-treated *Kras*$^{G12D/+}$;*p53*$^{\Delta/\Delta}$ lung tumour bearing mice. (d-e) Representative images (d) and quantification (e) of Ki67 positive cells. (f-g) Cleaved caspase-3 (cl. Casp3) staining (f) and quantification (g). Quantifications are represented as *Mcl-1* RNA positive signal per tumour area for (b), Ki67 positive area relative to area of the assessed lesions for (d), and cleaved Caspase 3 positive cell number to area of the lesions for (f). For data in panel c, e, and g six to eight lesions per section per mouse were analysed (at least 3 mice/group, n=number of lesions). Data in panel a, c, e, and g are presented as box plots, where the centre line represents the median value, the limits represent the 25th and 75th percentile, and the whiskers represent the minimum and maximum value of the distribution, excluding the outliers defined by the IQR rule.

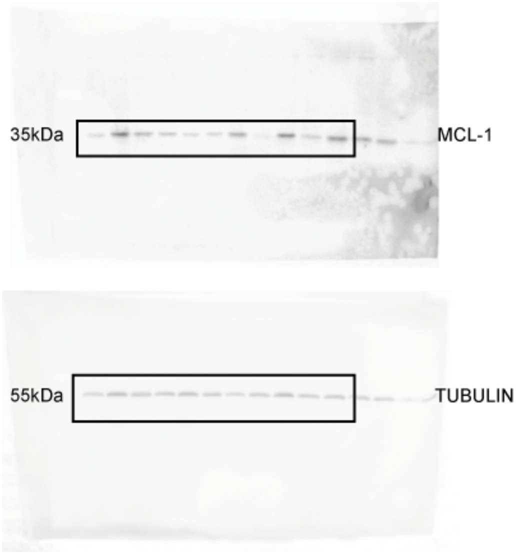

Supplementary Fig. 12
Full blots of the cropped images (black squares) reported in main Fig. 4a.

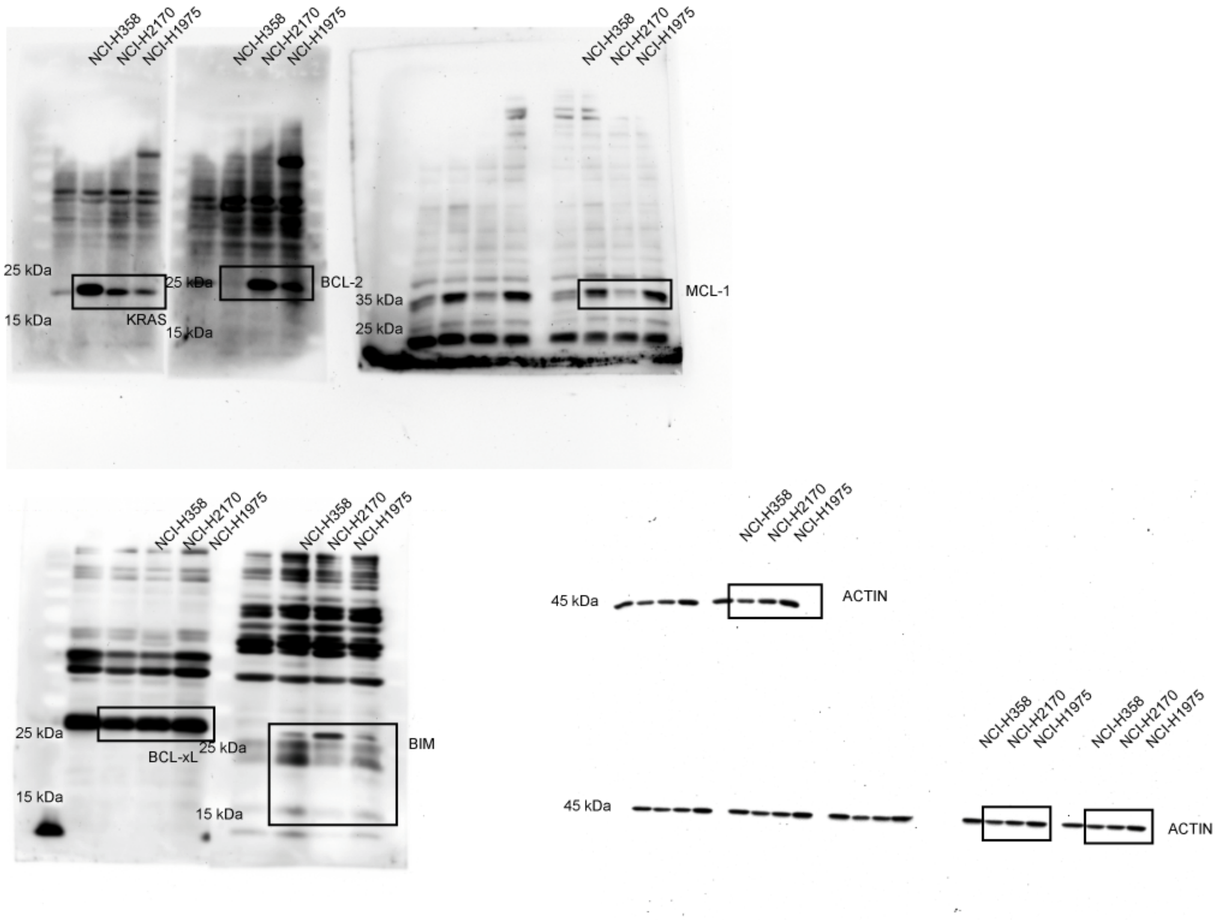

Supplementary Fig. 13
Full blots of the cropped images (black squares) reported in Supplementary Fig. 8c.

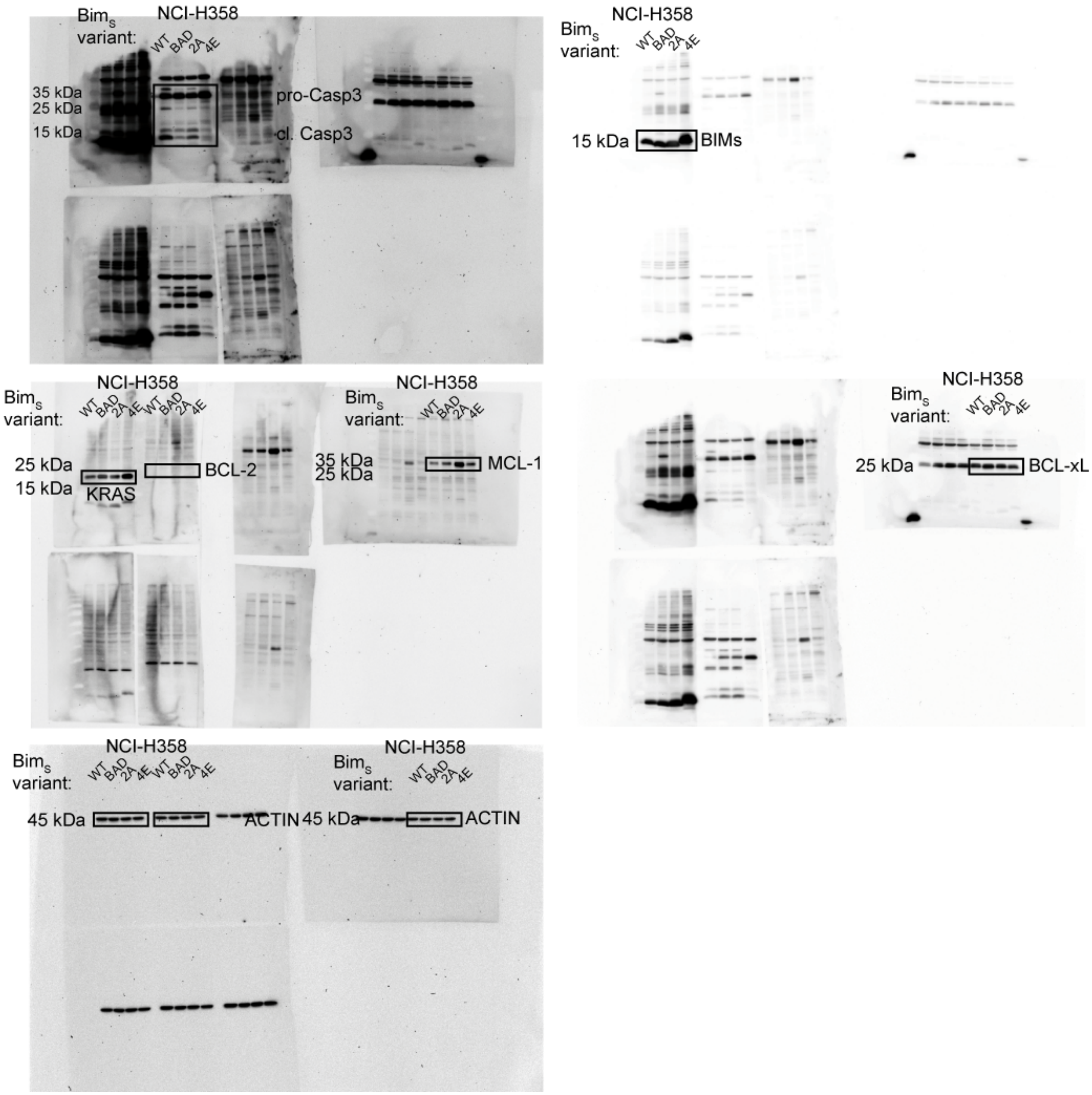

Supplementary Fig. 14

Full blots of the cropped images (black squares) reported for NCI-H358 in Supplementary Fig. 8f.

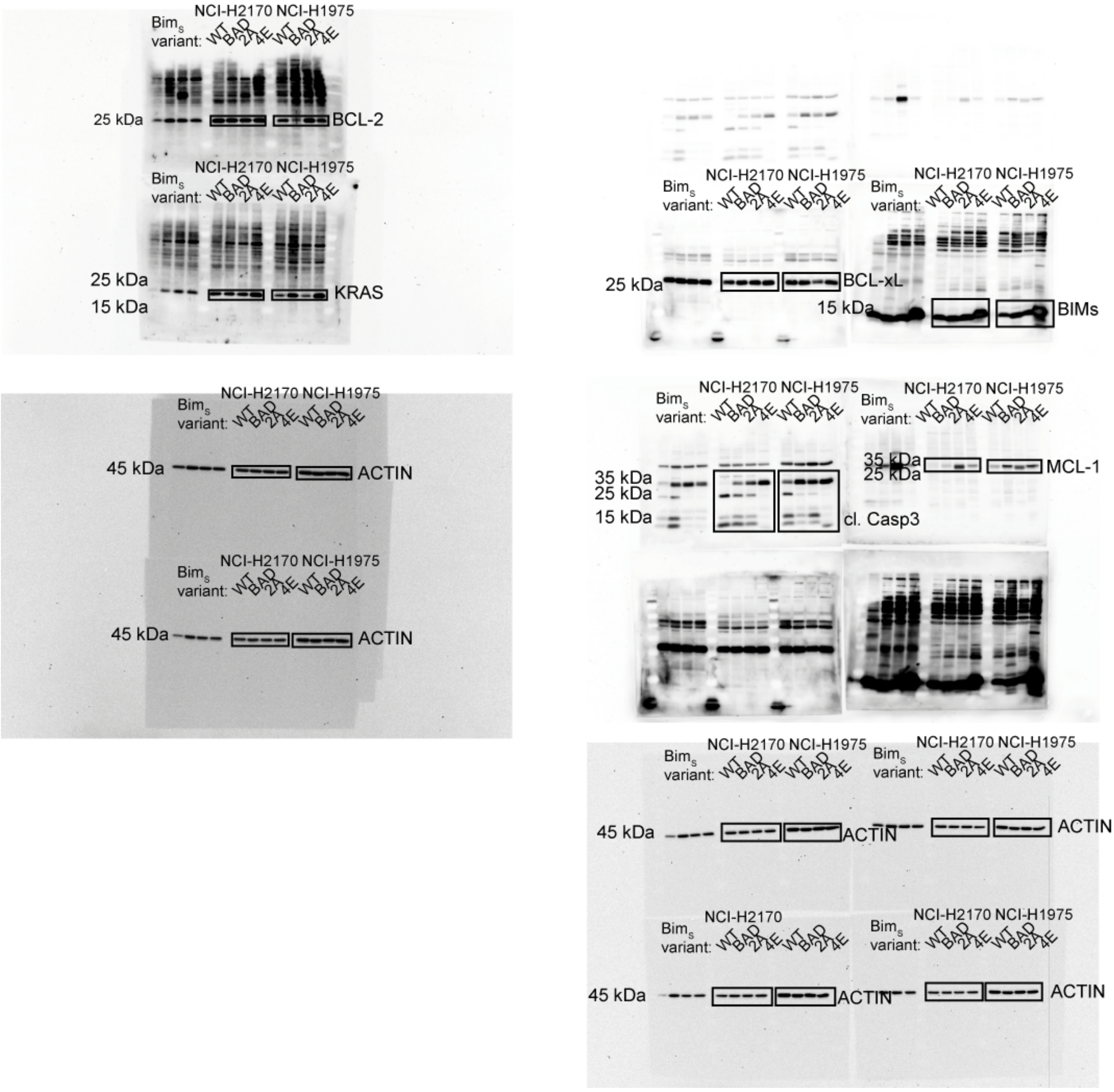

Supplementary Fig. 15

Full blots of the cropped images (black squares) reported for NCI-H1975 and NCI-H2170 in Supplementary Fig. 8f.

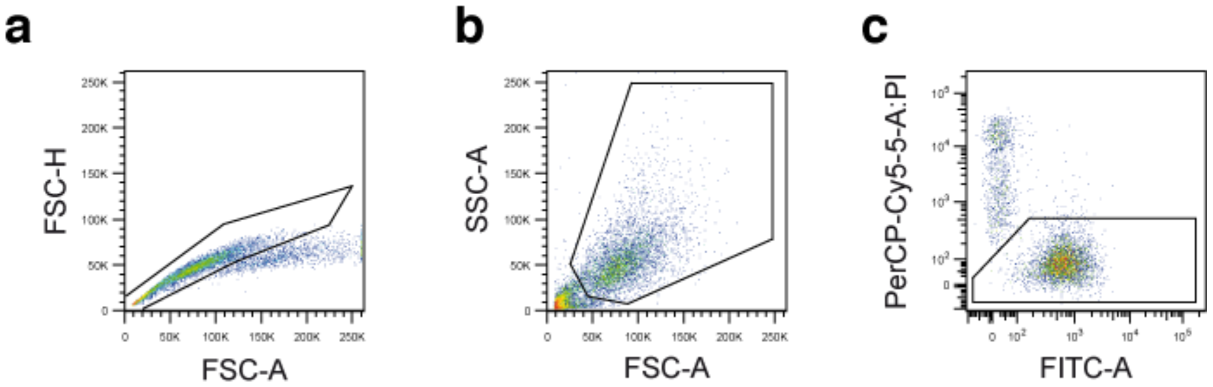

Supplementary Fig. 16 Gating strategy used for FACS analysis
(a) Pulse geometry gating — to remove doublets from the dataset. In the case of clumps of cells, the transit time increases, thus the area will also increase. In a plot of the area versus the height measurement, the single cells typically fall along a diagonal, while the clumps of cells will show up with increased area relative to the height. Using this pulse geometry gate removes these clumps, which is important because flow cytometry analysis is based on single cell analysis, not doublet cell analysis or 'clump' analysis. (b) Forward (FSC) versus Side Scatter (SSC) gating — to remove debris and other events of non-interest while preserving cells based on size and or complexity. The goal is to identify the cells of interest based on the relative size and complexity of the cells, while removing debris and other events that are not of interest. The gating strategy is as generous as possible, to eliminate only those events that are absolutely not of interest. The events with very low FSC and SSC, as well as those with low FSC and high SSC are eliminated. These events represent debris, cell fragments, and pyknotic cells. (c) Viability gating. Plotting a viability marker (propidium iodide, PI) against the FITC channel (GFP is stable expressed by the BIM_S variants) identifies the alive cells. PI is a DNA binding dye that is not permeable to intact membrane, meaning living cells will exclude the dye and exhibit little to no fluorescence.

Supplementary Tables

Supplementary Table 1: List of putative cancer genes that are located on the same amplicon as the *MCL-1* gene and show a significant increase in expression in samples with copy number gains versus samples with no gain. Bonferroni corrected p-values of one-sided Wilcoxon-test of expression values (tpm values) between TRACERx samples with high-level gain, low- level gain or no gain of the corresponding genes are reported in this table. Empty cells mean that this gene did not appear as significant in this cancer type. NA has been placed when no samples were present in the high gain group, thus only a test between gain and no gain could be conducted.

	**LUAD**			**LUSC**		
gene	low-level gain *vs* no gain	low-level gain *vs* high-level gain	high-level gain *vs* no gain	low-level gain *vs* no gain	low-level gain *vs* high-level gain	high-level gain *vs* no gain
MCL-1	0.59	0.022	0.0013	0.0057	NA	NA
MLLT11	0.019	0.22	0.0024			
ABL2	3.30E-05	NA	NA	0.00092	NA	NA
ARNT	1.20E-07	0.5	0.00071	0.0018	NA	NA
PRCC	2.10E-05	1	0.001	8.10E-10	NA	NA
RIT1	0.00053	0.052	1.00E-05	0.00094	NA	NA
BCL9	2.80E-05	1	0.007			
SDHC	0.0015	0.79	0.00072	3.50E-07	NA	NA
TPM3	0.088	0.019	9.50E-05	8.00E-04	NA	NA
TPR	6.00E-08	0.68	0.3	8.30E-05	NA	NA
CDC73	1.60E-08	NA	NA	0.00088	NA	NA
PDE4DIP	0.009	NA	NA			
SETDB1	1.70E-07	0.17	7.00E-07	0.00014	NA	NA
LMNA				0.0012	NA	NA
PBX1				0.044	NA	NA

Supplementary Table 2: List of samples used for the TMA. In this table we report: (i) ID of the sample; (ii) intensity of the staining for MCL-1, classified as strong, intermediate, and weak, as reported in main Fig. 3; and (iii) the results of the FISH analysis with *MCL-1* probes, classified as disomy, polysomy, low- or high-level gain (LLG or HLG, respectively).

	**ID sample**	**MCL-1 staining intensity**	***MCL-1* FISH analysis**
1	19149/00 II	strong	Disomy
2	3030/15 IA	medium	Polysomy
3	17084/13 IIA	weak	Polysomy
4	24345/07 IIIA	strong	Disomy
5	688/15 IC	strong	LLG
6	13962/15 IIB	weak	Disomy
7	18930/08 IIIC	strong	Disomy
8	21095/14 IIC	strong	Disomy
9	2116/15 A	strong	HLG
10	14202/01 A	strong	Polisomy
11	8150/01	strong	Polysomy
12	16437/05 IIIA	strong	Polysomy
13	4641/02 IB	medium	Disomy
14	7757/04 VB	medium	Polysomy
15	4791/04 IIIF	weak	Polysomy
16	7310/15 IB	medium	Disomy
17	1754/00	weak	Polysomy
18	11703/08 ID	medium	Disomy
19	398/13 IC	medium	LLG
20	11829/00 VE	medium	Disomy with focal Polysomy
21	17444/07 IA	medium	Polysomy
22	1848/07 IIIC	weak	Polysomy
23	7323/02 IIIA	strong	Polysomy
24	18971/02 IV A	medium	Disomy

Supplementary Table 3: Cell lines used in this study.

**#**		**ATCC**	**histological type**	***KRAS***	***TP53***
**1**	A549	ATCC® CCL-185™	NSCLC adenocarcinoma	+	
**2**	HCC44	ACC 534 (DSMZ)	NSCLC adenocarcinoma	+	+
**3**	NCI-H23	ATCC® CRL-5800™	NSCLC adenocarcinoma	+	+
**4**	NCI-H358	ATCC® CRL-5807™	NSCLC adenocarcinoma	+	
**5**	NCI-H1437	ATCC® CRL-5872™	NSCLC adenocarcinoma		+
**6**	NCI-H1650	ATCC® CRL-5883™	NSCLC adenocarcinoma		+
**7**	NCI-H1975	ATCC® CRL-5908™	NSCLC adenocarcinoma		+
**8**	NCI-H2087	ATCC® CRL-5922™	NSCLC adenocarcinoma		+
**9**	NCI-H2126	ATCC® CCL-256™	NSCLC adenocarcinoma		+
**10**	NCI-H2170	ATCC® CRL-5928™	NSCLC squamous		+
**11**	NCI-H1299	ATCC® CRL-5803™	NSCLC large cell		
**12**	NCI-H661	ATCC® HTB-183™	NSCLC large cell		+

Supplementary Table 4: The mRNA levels of the cancer genes identified in Supplementary Fig. 3c for the LUAD cell lines used in this study (normalised to actin and expressed as fold-change over A549).

		A549	HCC44	NCI-H23	NCI-H358	NCI-H1437	NCI-H1650	NCI-H1975	NCI-H2087	NCI-H2126
MCL-1	mean	1.013	1.080	4.132	2.457	6.233	0.972	1.435	1.453	6.636
	SD	0.200	0.297	0.351	0.208	0.519	0.229	0.473	0.200	0.187
MLLT11	mean	1.020	0.137	2.048	0.039	1.035	0.057	0.003	0.044	0.539
	SD	0.281	0.138	0.430	0.015	0.061	0.003	0.004	0.030	0.199
BCL9	mean	1.003	1.051	5.513	4.503	21.530	1.572	1.112	1.830	8.725
	SD	0.113	0.416	1.599	0.834	1.761	0.230	0.103	0.304	2.004
RIT1	mean	1.033	1.663	3.513	5.059	6.516	6.063	0.893	3.518	3.660
	SD	0.367	0.016	0.446	2.577	0.255	0.000	0.161	0.327	0.430
PRCC	mean	1.000	1.329	3.486	3.847	2.381	1.714	7.552	1.260	2.976
	SD	0.000	0.284	0.017	0.113	0.452	0.251	1.033	0.080	0.537
SETDB1	mean	1.000	1.887	1.771	3.938	7.867	3.240	0.712	1.016	3.005
	SD	0.044	0.157	0.156	0.665	0.312	0.863	0.031	0.050	0.715
ABL2	mean	1.002	0.666	0.978	2.385	2.474	0.964	0.365	0.897	2.080
	SD	0.083	0.029	0.134	0.105	0.108	0.228	0.025	0.157	0.051
ARNT	mean	1.003	0.678	1.461	2.233	7.648	0.774	0.501	0.767	3.062
	SD	0.113	0.040	0.007	0.273	0.133	0.208	0.042	0.019	0.845
SDHC	mean	1.001	1.083	2.187	1.539	2.507	1.790	1.193	1.488	5.751
	SD	0.064	0.069	0.182	0.388	0.375	0.760	0.023	0.087	0.451
TPM3	mean	1.000	1.807	4.456	4.974	8.092	0.463	1.874	2.184	4.513
	SD	0.044	0.044	0.480	0.268	0.999	0.129	0.092	0.054	1.015
TPR	mean	1.079	0.769	0.832	0.872	1.050	0.492	0.501	0.621	1.042
	SD	0.573	0.453	0.513	0.174	0.077	0.027	0.349	0.033	0.549
CDC73	mean	1.001	1.793	0.897	3.395	3.410	2.489	1.011	1.029	4.764
	SD	0.059	1.254	0.606	0.563	0.217	0.425	0.025	0.642	2.791
PDE4DIP	mean	1.062	1.862	18.450	0.002	0.253	0.484	0.001	0.013	0.014
	SD	0.411	0.468	2.442	0.000	0.136	0.370	0.000	0.013	0.023

Supplementary Table 5: R^2 and p values from simple linear regression between mRNA levels of the cancer genes and MCL-1 inhibitor sensitivity in the LUAD cell lines. No correction for multiple comparisons was applied. Cell viability after S63845 MCL-1 inhibitor treatment correlated significantly only with *MCL-1* mRNA levels.

	**p value**	**R^2**
ABL2	0.409	0.099
ARNT	0.384	0.110
BCL9	0.230	0.198
CDC73	0.412	0.098
MCL-1	**0.017**	**0.578**
MLLT11	0.140	0.283
PDE4DIP	0.080	0.373
PRCC	0.418	0.095
RIT1	0.399	0.103
SDHC	0.052	0.438
SETDB1	0.562	0.050
TPM3	0.095	0.346
TPR	0.695	0.023

Supplementary Table 6: *Mcl-1* copy number evaluated by Taqman® assay using the CopyCaller® Software. Primary cell cultures were generated as described in the Methods section from independent tumour lesions and were passaged over time to ensure the removal of fibroblast. This table also reports also the C_t value for the reference gene (*Tfrc*) used for the analysis (ND, not detectable).

sample	*Mcl-1* copy number	*Tfrc* C_t
sample 1	ND	29.3792
sample 2	ND	29.8119
sample 3	0.12	27.1841
sample 4	0.07	27.235
