# Supplementary material for: MCL-1 gains occur with high frequency in lung adenocarcinoma and can be targeted therapeutically
Source: Nat Commun. 2020 Sep 10;11:4527. doi: 10.1038/s41467-020-18372-1 (PMC7484793; doi:10.1038/s41467-020-18372-1)
Supplement: Supplementary file 3 — Description of Additional Supplementary Files [file 41467_2020_18372_MOESM3_ESM.pdf]

Description of Additional Supplementary Files

File Name: Supplementary Data 1
Description: List of the 316 genes in the 1q21 region (from 144854582 to 200960915), whose mRNA levels are significantly upregulated in TRACERx LUAD samples. The genes have been ranked by starting position. One-sided Wilcoxon testing without correction for multiple comparisons was used to identify significance and the corresponding p-values are reported in the table. In bold are reported the putative cancer genes.

File Name: Supplementary Data 2
Description: list of the 276 genes in the 1q21 region (from 144946769 to 200843305), whose mRNA levels are significantly upregulated in TRACERx LUSC samples. The genes have been ranked by starting position. One-sided Wilcoxon testing without correction for multiple comparisons was used to identify significance and the corresponding p-values are reported in the table. In bold are reported the putative cancer genes.
