# Supplementary material for: MCL-1 gains occur with high frequency in lung adenocarcinoma and can be targeted therapeutically
Source: Nat Commun. 2020 Sep 10;11:4527. doi: 10.1038/s41467-020-18372-1 (PMC7484793; doi:10.1038/s41467-020-18372-1)
Supplement: Supplementary file 6 — Reporting Summary [file 41467_2020_18372_MOESM6_ESM.pdf]

nature research

Corresponding author(s): Prof. Philipp Jost

Last updated by author(s): Aug 7, 2020

Reporting Summary

Nature Research wishes to improve the reproducibility of the work that we publish. This form provides structure for consistency and transparency in reporting. For further information on Nature Research policies, see our Editorial Policies and the Editorial Policy Checklist.

Statistics

For all statistical analyses, confirm that the following items are present in the figure legend, table legend, main text, or Methods section.

n/a	Confirmed	
☐	☒	The exact sample size (*n*) for each experimental group/condition, given as a discrete number and unit of measurement
☐	☒	A statement on whether measurements were taken from distinct samples or whether the same sample was measured repeatedly
☐	☒	The statistical test(s) used AND whether they are one- or two-sided<br>*Only common tests should be described solely by name; describe more complex techniques in the Methods section.*
☐	☒	A description of all covariates tested
☐	☒	A description of any assumptions or corrections, such as tests of normality and adjustment for multiple comparisons
☐	☒	A full description of the statistical parameters including central tendency (e.g. means) or other basic estimates (e.g. regression coefficient) AND variation (e.g. standard deviation) or associated estimates of uncertainty (e.g. confidence intervals)
☐	☒	For null hypothesis testing, the test statistic (e.g. *F*, *t*, *r*) with confidence intervals, effect sizes, degrees of freedom and *P* value noted<br>*Give P values as exact values whenever suitable.*
☒	☐	For Bayesian analysis, information on the choice of priors and Markov chain Monte Carlo settings
☒	☐	For hierarchical and complex designs, identification of the appropriate level for tests and full reporting of outcomes
☒	☐	Estimates of effect sizes (e.g. Cohen's *d*, Pearson's *r*), indicating how they were calculated

Our web collection on statistics for biologists contains articles on many of the points above.

Software and code

Policy information about availability of computer code

Data collection	The data collection and processing were performed in the R statistical environment version > = 3.3.1.
Data analysis	Code for the data analysis can be downloaded from https://github.com/McGranahanLab/MCL1amplification

For manuscripts utilizing custom algorithms or software that are central to the research but not yet described in published literature, software must be made available to editors and reviewers. We strongly encourage code deposition in a community repository (e.g. GitHub). See the Nature Research guidelines for submitting code & software for further information.

Data

Policy information about availability of data

All manuscripts must include a data availability statement. This statement should provide the following information, where applicable:

- Accession codes, unique identifiers, or web links for publicly available datasets
- A list of figures that have associated raw data
- A description of any restrictions on data availability

The data that support the findings of this study are available from the corresponding author upon reasonable request. The data generated by The Cancer Genome Atlas pilot project established by the NCI and the National Human Genome Research Institute was downloaded. The data were retrieved through database of Genotypes and Phenotypes (dbGaP) authorisation (accession no. phs000854.v3.p8 [https://www.ncbi.nlm.nih.gov/projects/gap/cgi-bin/study.cgi?study_id=phs000178.v11.p8]). Information about TCGA and the investigators and institutions who constitute the TCGA research network can be found at https://cancergenome.nih.gov/. The genomic TRACERx data can be downloaded from the European Genome-Phenome Archive (EGA), which is hosted by the European Bioinformatics Institute (EBI) and the Centre for Genomic Regulation (CRG), under the accession number EGAS00001002247 [https://www.ebi.ac.uk/ega/studies/EGAS00001002247].

Field-specific reporting

Please select the one below that is the best fit for your research. If you are not sure, read the appropriate sections before making your selection.

☒ Life sciences ☐ Behavioural & social sciences ☐ Ecological, evolutionary & environmental sciences

For a reference copy of the document with all sections, see nature.com/documents/nr-reporting-summary-flat.pdf

Life sciences study design

All studies must disclose on these points even when the disclosure is negative.

Sample size	Our sample sizes was estimated in line with previously published papers (such as PMID: 24326876 and 29370714). With this sample size, each experiment had 80% power to detect a Cohen's effect size of 1.5 for each pairwise comparison. This calculation was for a significance level of 0.05 without correction for multiple comparisons.
Data exclusions	No data were excluded from the analysis
Replication	Genomic analysis was confirmed in three different independent cohorts.<br>Every in vitro experiment was performed in replicates in at least 3 independent experiments. All the attempts at replication were successful.<br>For the in vivo experiments reported in Fig. 5 mice were infected in 4 different cohorts balanced by genotype. For the IHC quantification different lesions/slides per animal were evaluated (as reported in figure legends). All the attempts at replication were successful.
Randomization	Human cases were selected randomly among the tissue available.<br>For the in vivo experiments reported in Fig. 5 and 6, mice were assigned to a specific group based on their genotype after birth and there were no other factors that determined group selection. No mice were excluded.<br>For the in vitro experiments cells were plated in 96 well plates, and wells were assigned randomly to the different treatments.
Blinding	For the genomic analyses (reported in Fig. 1 and 2 and Supplementary Fig. 2-7) the experimenters were not blinded to the group allocation.<br>For the quantification of MCL-1 ICH and FISH on human tissue (reported in Fig. 3), the experimenters were blinded to the grade (Fig. 3a-e) or the diagnosis (Fig. 3g-h, healthy vs LUAD). For the in vitro experiments (reported in Fig. 4) the experimenters were blinded to the treatment (Fig. 4b and d) and the plasmid (Fig. 4e and Supplementary Fig. 8c-e).<br>For the quantification of the staining on mouse tissue (reported in Fig. 5b-e, Supplementary Fig.9-11) , the experimenters were blinded to the genotype (Fig.5b-e and Supplementary Fig. 9-10) or the treatment (Supplementary Fig. 11) .<br>For the evaluation of the micro CT analysis (reported in Fig. 5f-g and Fig. 6c-d), the experimenters were blinded to the genotype (Fig. 5f-g) or the treatment (Fig. 6c-d)

Reporting for specific materials, systems and methods

We require information from authors about some types of materials, experimental systems and methods used in many studies. Here, indicate whether each material, system or method listed is relevant to your study. If you are not sure if a list item applies to your research, read the appropriate section before selecting a response.

Materials & experimental systems

n/a	Involved in the study	
☐	☒	Antibodies
☐	☒	Eukaryotic cell lines
☒	☐	Palaeontology and archaeology
☐	☒	Animals and other organisms
☐	☒	Human research participants
☒	☐	Clinical data
☒	☐	Dual use research of concern

Methods

n/a	Involved in the study	
☒	☐	ChIP-seq
☐	☒	Flow cytometry
☒	☐	MRI-based neuroimaging

Antibodies

Antibodies used	The following antibodies were used:<br>alfa-tubulin (Cell Signaling, cat#9099)<br>beta-Actin (Cell Signaling, cat #4970, clone #13E5),<br>BCL-2 (Bcl-2-100, produced at WEHI),<br>BCL-XL (BD Bioscience, cat# 610212),<br>BIM (Enzo Life Science, cat# ADI-AAP-33-E),<br>Caspase-3 (Cell Signaling, cat# 9662),<br>CC10 (T18) (Santa-Cruz, cat #sc-9772),<br>cleaved caspase-3 (Asp175) (Cell Signaling, cat# 9661),<br>Ki67 (ThermoFisher, cat #RM-9106, clone #SP6),

	KRAS (SigmaAldrich, cat #MABS194, clone# 234-4.2),<br>MCL-1 (19C4-15, rat monoclonal, gift from David Huang, WEHI),<br>MCL-1 (Proteintech, cat #16225-1-AP),<br>SP-C (C19) (Santa-Cruz, cat #sc-7705).
Validation	For the commercially available antibodies, we relied on the data-sheet information and validation, specifically:<br>alfa-tubulin (Cell Signaling, cat#9099), this antibody is validated only for Western blot and it reacts against the human protein.<br>beta-Actin (Cell Signaling, cat #4970, clone #13E5), this antibody has been validated for WB and for specificity against the human protein.<br>BCL-XL (BD Bioscience, cat# 610212), this antibody is routinely tested for WB and the reactivity against human is used as quality control of every batch.<br>BIM (Enzo Life Science, cat# ADI-AAP-33-E), Bim is a group of three splice variants, BimEL, BimL, and BimS, with apparent molecular masses of ~23, 16, and 13 kDa, respectively; this antibody has been validated for WB and for specificity against human.<br>Caspase-3 (Cell Signaling, cat# 9662), this antibody has been validated for WB and for specificity against the human protein (using also KO cell line).<br>CC10 (T18) (Santa-Cruz, cat #sc-9772), epitope mapping near the C-terminus of CC10 of mouse origin, specific for mouse protein and validate in IHC.<br>cleaved caspase-3 (Asp175) (Cell Signaling, cat# 9661), this antibody cross-react with human and mouse samples and has been validated both for Western blot and IHC.<br>Ki67 (ThermoFisher, cat #RM-9106, clone #SP6), this antibody is recommended for immunohistochemistry.<br>KRAS (SigmaAldrich, cat #MABS194, clone# 234-4.2), this antibody is suited for western blot and is human-specific.<br>MCL-1 (Proteintech, cat #16225-1-AP), this antibody has been tested for western blot and immunohistochemistry and western blot both for human and mouse samples.<br>SP-C (C19) (Santa-Cruz, cat #sc-7705), has been used for detection of SP-C in mouse lung in PMID 21743024, 17476360 and 24430801.<br>For the antibodies produced at WEHI, the validation can be found in the following paper: PMID 15694340.

Eukaryotic cell lines

Policy information about cell lines

Cell line source(s)	Human NSCLC cell lines: A549, NCI-H23, NCI-H358, NCI-H1437, NCI-H1650, NCI-H1975, NCI-H2087, NCI-H2126, NCI-H661, NCI-H1299, and NCI-H2170 were obtained from ATCC and HCC44 was purchased from Leibniz Institute DSMZ.
Authentication	The human NSCLC cell lines A549, NCI-H23, NCI-H358, NCI-H1437, NCI-H1650, NCI-H1975, NCI-H2087, NCI-H2126, NCI-H661, NCI-H1299, and NCI-H2170 were authenticated with STR profiling analysis by ATCC. HCC44 was analysed with STR profiling and verified by DSMZ profile database
Mycoplasma contamination	All the cell lines used for this study resulted negative for mycoplasma contamination.
Commonly misidentified lines<br>(See ICLAC register)	For the presented study, no commonly misidentified cell lines were used.

Animals and other organisms

Policy information about studies involving animals; ARRIVE guidelines recommended for reporting animal research

Laboratory animals	mus musculus, C57bl, females and males, from 6-8 week of age until 25-27 week of age.
Wild animals	For the presented study, no wild animals were used.
Field-collected samples	For the presented study, no field-collected samples were used.
Ethics oversight	All the animal experiments were conducted in compliance with protocols approved by the District Government of Upper Bavaria in line with the animal ethics committee guidelines (Az: 55.2-1-54-2532- 55-12 and ROB-55.2-2532.Vet_02-14-69.).

Note that full information on the approval of the study protocol must also be provided in the manuscript.

Human research participants

Policy information about studies involving human research participants

Population characteristics	For the TMAs, only anonymised materials from the archives of pathology were used.<br>For the TRACERx dataset, these data have been already reported in PMID: 28445112.
Recruitment	For the TMAs, all samples of a respective tumour region were extracted from areas exhibiting a high tumour/stroma ratio. Obvious inflammatory hotspots (such as lymph follicles or areas of ulceration) were avoided.<br>For the TRACERx dataset, please refer to PMID 28445112 and the specific clinical trials C11496/A17786 and NCT01888601.
Ethics oversight	All the studies were conducted in accordance with the World Medical Association Declaration of Helsinki entitled 'Ethical Principles for Medical Research Involving Human Subjects' (1996 version).<br>For the TMAs: the first TMA has been approved by the human ethics committee from the Thoraxklinik Heidelberg, Germany (no. 206/2005). The second TMA was generated from the archive of the Institute of Pathology of the TUM and provided by the MRI/TUM-biobank (approved by ethical committee Nr. 553/15 S). All patients had given written consent to the use of

surgically removed tissue for scientific purposes.
The TRACERx study was conducted under the favourable opinion from the NRES Committee London – Camden & Islington Research Ethics Committee.

Note that full information on the approval of the study protocol must also be provided in the manuscript.

Flow Cytometry

Plots

Confirm that:

☒ The axis labels state the marker and fluorochrome used (e.g. CD4-FITC).

☒ The axis scales are clearly visible. Include numbers along axes only for bottom left plot of group (a 'group' is an analysis of identical markers).

☒ All plots are contour plots with outliers or pseudocolor plots.

☒ A numerical value for number of cells or percentage (with statistics) is provided.

Methodology

Sample preparation	For experiments reported in Fig. 4e and Supplementary Fig. 8d after the indicated amount of time cells were collected in FACS buffer (PBS+3%FBS). Single cell suspensions were stained with 3µg/ml propidium iodide.
Instrument	Data were acquired on BD FACS Canto II (BD Biosciences)
Software	Data data were analysed using FlowJo software (Tree Star).
Cell population abundance	FACS analysis was used to determine cell viability and not to select a specific population.
Gating strategy	1. Pulse geometry gating — to remove doublets from the dataset. In the case of clumps of cells, the transit time increases, thus the area will also increase. In a plot of the area versus the height measurement, the single cells typically fall along a diagonal, while the clumps of cells will show up with increased area relative to the height. Using this pulse geometry gate removes these clumps, which is important because flow cytometry analysis is based on single cell analysis, not doublet cell analysis or 'clump' analysis.<br>2. Forward (FSC) versus Side Scatter (SSC) gating — to remove debris and other events of non-interest while preserving cells based on size and or complexity. The goal is to identify the cells of interest based on the relative size and complexity of the cells, while removing debris and other events that are not of interest. The gating strategy is as generous as possible, to eliminate only those events that are absolutely not of interest. The events with very low FSC and SSC, as well as those with low FSC and high SSC are eliminated. These events represent debris, cell fragments, and pyknotic cells.<br>3. Viability gating. Plotting a viability marker (propidium iodide, PI) against the FITC channel (GFP is stable expressed by the BIMS variants) identifies the alive cells. PI is a DNA binding dye that is not permeable to intact membrane, meaning living cells will exclude the dye and exhibit little to no fluorescence.

☒ Tick this box to confirm that a figure exemplifying the gating strategy is provided in the Supplementary Information.
